# Contrast Enema: Solving Diagnostic Dilemmas in Neonates With Lower Intestinal Obstruction

**DOI:** 10.7759/cureus.23458

**Published:** 2022-03-24

**Authors:** Anum Manzoor, Nabila Talat, Hafiz Muhammad Adnan, Muhammad W Zia, Muhammad Ahsen Aziz, Ezza Ahmed

**Affiliations:** 1 Pediatric Surgery, The Children’s Hospital and University of Child Health Sciences, Lahore, PAK

**Keywords:** hirschsprung’s disease, ileal atresia, meconium ileus, intestinal obstruction, contrast enema

## Abstract

Background

Anatomical abnormalities leading to bowel movement failure are the major cause of intestinal obstruction. This study was done to assess the diagnostic efficacy of contrast enema in neonates with lower intestinal obstruction.

Methodology

This prospective study was conducted in The Children’s Hospital and University of Child Health Sciences, Lahore from February 2021 to July 2021. Patients presenting with constipation, abdominal distension, vomiting, and unable to pass meconium, evaluated clinically and by X-rays, were included in the study. Patients with lower intestinal obstruction (i.e., meconium ileus, Hirschsprung’s disease, ileal atresia, meconium plug syndrome, and small left colon) were given contrast enema (gastrografin) after hydration. The radiologist was blinded by the clinical diagnosis or reference standard diagnosis, which was labeled prior to image interpretation. Sensitivity, specificity, positive predictive value (PPV), and negative predictive value (NPV) were calculated for major contrast enema diagnosis.

Results

During this period, a total of 34 patients were included in the study. The mean age of presentation was 72.0 ± 24 hours. There were 21 (61.8%) boys and 13 (38.2%) girls, representing a male to female ratio of 1.7:1. In four cases, our diagnosis on the basis of contrast enema was proven wrong. Sensitivity, specificity, PPV, and NPV of Hirschsprung’s disease were found to be 93.3%, 50%, 87.5%, and 66.7%, while these were 84.6%, 66.7%, 91.7%, and 50.0%, respectively, for meconium ileus.

Conclusion

The diagnostic efficacy of contrast enema was found to be good in neonates with lower intestinal obstruction. The spectrum of intestinal obstruction among neonates shows diversity while contrast enema seems to play a major role in the identification and remodeling of the diagnostic plan in a major proportion of cases.

## Introduction

Neonatal intestinal obstruction is caused by an anatomical abnormality that leads to bowel movement failure [1]. Neonates are the most commonly affected age group for intestinal obstruction and these obstructions are usually categorized as high obstruction or low obstruction, depending upon the radiological findings. The main causes of low intestinal obstruction are anorectal malformation, ileal atresia, Hirschsprung's disease (HD), meconium ileus (MI), and meconium plug syndrome [2]. Intestinal atresia is one of the leading causes of neonatal intestinal obstruction. The most common sites are the ileum [3]. MI is another common cause of low intestinal obstruction and is generally considered one of the major initial signs of cystic fibrosis. Around 20% of cases of cystic fibrosis have MI [4]. HD is also a common cause of intestinal obstruction in neonates [5].

Apart from anorectal malformations, which are diagnosed clinically, exact diagnosis of the cause of low intestinal obstruction on the basis of clinical examination and plain X-ray is difficult. Contrast enema (CE) is considered to narrow down the differential diagnosis and may go on to recognize abnormalities like MI, small intestinal atresia (SIA), colonic atresia, small left colon syndrome, and HD [6]. Many types of contrasts are available but the most commonly used is gastrografin because it has both diagnostic and therapeutic roles.

An exact preoperative diagnosis can change the management plan of the patient [6,7], i.e., MI can be managed medically and HD can be managed in single-stage surgery. A better understanding of the diagnostic modalities related to intestinal obstruction is important. Computed tomography (CT) and plain or contrast imaging modalities can be of real help in diagnosing the possible causes of intestinal obstruction [8-10]. We conducted this study to assess the diagnostic efficacy of CE in neonates with lower intestinal obstruction as not many previous prospective studies were available.

## Materials and methods

Study design, place, and duration of the study

A prospective study was done at The Children’s Hospital and University of Child Health Sciences, Lahore, Pakistan from February 2021 to July 2021.

Inclusion and exclusion criteria

Patients presenting with abdominal distension, vomiting, and failure to pass meconium, evaluated clinically as well as by X-rays, were included in the study. Patients with signs of peritonitis or septicemia were excluded from the study.

Data collection

Approval from the Institutional Ethical Committee of The Children’s Hospital and University of Child Health Sciences, Lahore, Pakistan was acquired (letter number: CHICH/20-325). Informed and written consents were sought from parents/caregivers of all study participants. Patients with lower intestinal obstruction (i.e., meconium ileus, Hirschsprung’s disease, ileal atresia, meconium plug syndrome, and small left colon) were given contrast enema (gastrografin) after hydration. Foley's catheter was inserted rectally and a balloon was inflated (balloon was not inflated to its full capacity, only 0.5 cc to 1 cc of distilled water was used) to avoid leakage of contrast. Under gentle pressure, gastrografin was injected through Foley's catheter until the resistance to further injection was felt. Then Foley's catheter was clamped and X-rays were taken. Foley's catheter was removed after deflation of the bulb. The radiologist was blinded by the clinical diagnosis or reference standard diagnosis, which was labeled prior to image interpretation to get an unbiased view. All the study data were designed on a pre-designed proforma.

Statistical analysis

The data analysis was performed by Statistical Package for the Social Sciences (SPSS) version 26.0 (IBM Corp., Armonk, NY). Qualitative data were represented as frequency and percentages whereas quantitative variables were shown as mean ± standard deviation (SD). Sensitivity, specificity, positive predictive value (PPV), and negative predictive value (NPV) were calculated for each contrast enema diagnosis.

## Results

During this period, a total of 34 patients were included in the study. The mean age of presentation was 72.0 ± 24 hours. There were 21 (61.8%) boys and 13 (38.2%) girls, representing a male to female ratio of 1.7:1. In 30 patients, the post-contrast diagnosis was accurate and the management plan based on post-contrast diagnosis was successful. Table 1 shows the details of these 30 patients with pre-contrast diagnosis, decided plan, post-contrast diagnosis, and post-contrast plan. The diagnosis was made on the basis of contrast enema and management plans were changed. In eight patients, a pre-contrast diagnosis of non-specific intestinal obstruction was made and the plan was the exploration of patients; post-contrast, the diagnosis turned out to be meconium ileus and the patients were managed medically and were saved from surgery. In 10 patients, a pre-contrast diagnosis of HD was changed to short segment HD after contrast, and plans of laparotomy and biopsies were changed to sigmoid loop colostomy and biopsy. In seven patients, a pre-contrast diagnosis of intestinal obstruction was changed to short segment HD after contrast. The plan was changed from exploration to non-operative management, and the patients were prepared for single-stage surgery. In one patient, the diagnosis was changed from intestinal obstruction to small left colon syndrome and the patient was managed non-operatively.

**Table 1:** Patients in whom the post-contrast diagnosis was correct (n = 30).

Number of patients	Pre-contrast diagnosis	Plan	Post-contrast diagnosis	Post-contrast plan
8	Non-specific intestinal obstruction	Exploratory laparotomy	Meconium Ileus	Conservative management
6	Hirschsprung's disease	Exploratory laparotomy + biopsy	Short segment Hirschsprung's disease	Sigmoid loop colostomy + biopsy
7	Intestinal obstruction	Exploratory laparotomy	Short segment Hirschsprung's disease	Sigmoid loop colostomy + biopsy
1	Intestinal obstruction	Exploratory laparotomy	Small left colon syndrome	Conservative management
4	Intestinal atresia	Exploratory laparotomy	Ileal atresia	Exploratory laparotomy
3	Hirschsprung's disease	Exploratory laparotomy	Long segment Hirschsprung's disease	Exploratory laparotomy + biopsy
1	Intestinal obstruction	Exploratory laparotomy	Complicated meconium ileus + meconium cyst	Exploratory laparotomy

Overall, contrast enema’s diagnosis was matched with 30/34 (88.2%) cases following reference standard diagnosis. In four cases, our diagnosis on the basis of contrast enema was wrong and the management plan based on post-contrast diagnosis was not successful. Table 2 shows the details of these four patients in whom the diagnosis on the basis of contrast enema was proven wrong. In one of the patients, a post-contrast diagnosis of meconium ileus was made but it turned out to be a case of ileal atresia. In the second patient, a post-contrast diagnosis of meconium ileus was made but it turned out to be a ruptured meconium cyst. In the third patient, a post-contrast diagnosis of Hirschsprung's disease was made but it turned out to be ascending colon atresia. In the fourth patient, a post-contrast diagnosis of Hirschsprung's disease was made and it turned out to be necrotizing enterocolitis. Table 3 shows the sensitivity, specificity, PPV, and NPV of contrast enema with respect to standard reference diagnosis. Sensitivity, specificity, PPV, and NPV of Hirschsprung’s disease were found to be 93.3%, 50%, 87.5%, and 66.7%, while these were 84.6%, 66.7%, 91.7%, and 50.0%, respectively, for meconium ileus.

**Table 2:** Details of patients in whom the post-contrast diagnosis was wrong (n = 4).

Number of patients	Post-contrast diagnosis	Management plan	Outcome of the management plan	Surgical management done	Final diagnosis after surgery
1	Meconium ileus	Conservative management	Failed	Exploratory laparotomy	Ileal atresia
1	Meconium ileus	Conservative management	Failed	Exploratory laparotomy	Ruptured meconium cyst
1	Hirschsprung's disease	Conservative management	Failed	Exploratory laparotomy	Ascending colon atresia
1	Hirschsprung's disease	Conservative management	Failed	Exploratory laparotomy	Necrotizing enterocolitis

**Table 3:** Sensitivity, specificity, positive predictive value, and negative predictive value of contrast enema with respect to standard reference diagnosis.

Diagnosis	Sensitivity	Specificity	Positive predictive value	Negative predictive value
Hirschsprung's disease	93.3%	50%	87.5%	66.7%
Meconium ileus	84.6%	66.7%	91.7%	50.0%

## Discussion

In our study, we evaluated the diagnostic performance of CE across a range of diagnoses that are usually encountered in neonates. Not many studies in the past have been done to elaborate the diagnostic effectiveness of CE aiming diagnosis of lower intestinal obstruction among neonates. It can be clinically difficult to distinguish small left colon syndrome from a completely unrelated meconium ileus. Traditional radiographic studies reveal distal bowel obstruction [11-13].

We found that CE diagnosis matched with 88.2% of cases following reference standard diagnosis. Findings of HD on a contrast enema study include an abnormal recto-sigmoid ratio < 1 and a transition zone of colonic narrowing [14]. The level of the transition zone can help to plan management. In this study, sensitivity, specificity, PPV, and NPV of CE for meconium ileus were noted to be 84.6%, 66.7%, 91.7%, and 50.0%, respectively. The literature has reported sensitivity and specificity of contrast enema in ileus diagnosis as 89.2% and 96.6% [15]. The researchers have reported HD to be the most common cause of intestinal obstruction in neonates and it was reiterated in the present research as well [16]. We found good sensitivity (93.3%) and PPV rates (87.5%) of CE in HD, which has been described by other researchers as well. In their study, De Lorijn et al. revealed sensitivity rates of rectal suction biopsy, anorectal manometry, and CE among HD cases to be 93%, 83%, and 76%, respectively, whereas specificity rates of rectal suction biopsy, anorectal manometry, and contrast exam were noted to be 100%, 93%, and 97%, respectively. Overall, De Lorijn et al. found no statistically significant difference in sensitivity and specificity rates among different diagnostic modalities in cases of HD [17].

Limitations of the study

Our study had some limitations as well. As this was a single-center study conducted on relatively small sample size, our findings cannot be generalized. The diagnosis of small left colon syndrome is primarily an imaging diagnosis and complicated by the original interpretation, which may affect the reference standard diagnosis. The radiologists were blinded to clinical information and interpretation was made primarily on the imaging appearance, while some of the clinical information is usually known prior to the exam.

## Conclusions

The diagnostic efficacy of contrast enema was found to be good in neonates with lower intestinal obstruction. The spectrum of intestinal obstruction among neonates shows diversity, while CE seems to play a major role in the identification and remodeling of the diagnostic plan in a major proportion of cases.
